# Insulin-Like Peptide Receptor-Mediated Signaling Pathways Orchestrate Regulation of Growth in the Pacific Oyster (*Crassostrea gigas*), as Revealed by Gene Expression Profiles

**DOI:** 10.3390/ijms22105259

**Published:** 2021-05-17

**Authors:** Yongjing Li, Huiru Fu, Fuqiang Zhang, Liting Ren, Jing Tian, Qi Li, Shikai Liu

**Affiliations:** 1 Key Laboratory of Mariculture (Ocean University of China), Ministry of Education, and College of Fisheries, Ocean University of China, Qingdao 266003, China; liyongjing@stu.ouc.edu.cn (Y.L.); fuhuiru@stu.ouc.edu.cn (H.F.); zhangfuqiang@stu.ouc.edu.cn (F.Z.); renliting@stu.ouc.edu.cn (L.R.); tj2357@stu.ouc.edu.cn (J.T.); szl0021@auburn.edu (Q.L.); 2 Laboratory for Marine Fisheries Science and Food Production Processes, Qingdao National Laboratory for Marine Science and Technology, Qingdao 266237, China

**Keywords:** *Crassostrea gigas*, IIS, nutrient abundance, temperature, energy metabolism

## Abstract

The involvement of insulin/insulin-like growth factor signaling (IIS) pathways in the growth regulation of marine invertebrates remains largely unexplored. In this study, we used a fast-growing Pacific oyster (*Crassostrea gigas*) variety “Haida No.1” as the material with which to unravel the role of IIS systems in growth regulation in oysters. Systematic bioinformatics analyses allowed us to identify major components of the IIS signaling pathway and insulin-like peptide receptor (ILPR)-mediated signaling pathways, including PI3K-AKT, RAS-MAPK, and TOR, in *C. gigas*. The expression levels of the major genes in IIS and its downstream signaling pathways were significantly higher in “Haida No.1” than in wild oysters, suggesting their involvement in the growth regulation of *C. gigas*. The expression profiles of IIS and its downstream signaling pathway genes were significantly altered by nutrient abundance and culture temperature. These results suggest that the IIS signaling pathway coupled with the ILPR-mediated signaling pathways orchestrate the regulation of energy metabolism to control growth in Pacific oysters.

## 1. Introduction

Nutrient abundance is one of the most important environmental factors that are critical to the growth and reproduction of all organisms. Nutrient sensing and energy metabolism are coordinated by networks of signaling cascades, such as the insulin/insulin-like growth factor signaling (IIS), target of rapamycin (TOR), and adenosine monophosphate-activated protein kinase (AMPK) signaling pathways [1]. The IIS and AMPK signaling pathways are two critical coordinators of energy homeostasis and metabolic processes across diverse vertebrate and invertebrate species. The TOR signaling pathway integrates various environmental factors and the signal transduction from the IIS and AMPK pathways in order to direct cell growth [1,2].

As one of the most important nutrient sensors, the mechanism of the IIS pathway in the growth regulation of vertebrates has been well studied. The IIS signaling pathway—including insulin/insulin-like growth factors (IGFs), insulin/IGF receptors (INSR, ILPR, and IGFR), insulin receptor substrate (IRS), insulin-like growth factor-binding proteins (IGFBPs), and insulin-like growth factor-binding protein complex acid labile subunits (IGFALSs)—regulates multiple cellular processes, including cell growth and proliferation, energy metabolism, hormone secretion, and glucose homeostasis [3,4,5]. In vertebrates, the insulin acutely alters in response to nutrient abundance and other circulating factors, in order to regulate the nutrient uptake and storage activity of organisms [6]. The IGFs are regulated by the growth hormone (GH), which is secreted by the neuroendocrine cells of the anterior pituitary gland, and function to regulate the growth of organisms [7,8]. The levels of IGFs in serum are strictly controlled by the ternary complex “IGF–IGFBP–ALS” [9]. Binding of insulin or IGFs to the tyrosine kinase receptors (INSR, IGFR) leads to the activation of the receptors and the phosphorylation of insulin receptor substrates (IRS), which cooperate to trigger the PI3K-AKT and RAS-MAPK pathways to regulate cell proliferation, glycogen metabolism, and protein synthesis [4,10].

Only insulin-like peptides, but no growth hormone, have been identified in various invertebrates [11,12,13]. The insulin-like peptides were produced from brain cells or small clusters of neuroendocrine cells, and were involved in neuroendocrine activity in several insects and gastropod mollusks [14,15,16,17,18]. The insulin-like peptide receptors and various IGFBPs in invertebrates, which share similar structures and functions with their counterparts in vertebrates, have also been identified [19,20,21,22,23,24,25,26,27]. Furthermore, genes involved in the PI3K-AKT and RAS-MAPK signaling pathways, such as PI3K, AKT, PTEN, and RAS, were also identified in invertebrates [28,29,30]. Levels of expression or phosphorylation of key components of these pathways have been associated with growth difference [29,31,32,33,34,35]. A recent study on pearl oysters reported that the insulin-like peptide recombinant protein induced the expression of ILPR and the major genes of the PI3K-AKT and RAS-MAPK pathways, suggesting a functional IIS signaling cascade mediated by ILPR [36]. Therefore, systemic characterization of IIS signaling and its downstream pathways that are involved in growth would be essential for unravelling the molecular mechanisms of growth regulation in invertebrates such as mollusks.

The Pacific oyster (*Crassostrea gigas*) is one of the most widely cultured marine mollusks, and has been introduced from Asia to many other countries around the world [37]. A selection breeding program of *C. gigas* in China performed for over ten years has produced a fast-growing variety named “Haida No.1”, which exhibits significant growth advantages over wild oysters [38,39]. The fast-growing variety provides an ideal research system for growth studies of oysters. In a previous work, we identified four insulin-like peptide genes in *C. gigas*, and showed that expression levels of *ILPs* were significantly higher in fast-growing “Haida No.1” than in wild oysters [40]. Environmental factors such as nutrient abundance and ambient temperature had significant effects on the expression of *ILP* genes and on the growth of the oysters. To further understand the role of the IIS signaling pathway in the growth regulation of oysters, in this work, we performed extensive multi-omics data mining in order to identify the key components of the IIS, PI3K-AKT, and RAS-MAPK signaling pathways in *C. gigas*. The expression profiles of the major genes of the IIS and ILPR-mediated signaling pathways were determined in “Haida No.1” and in wild oysters. Fasting/re-feeding and low-temperature culture experiments were performed in order to further understand how the IIS signaling pathway was altered by nutrient levels and temperature to affect the growth of *C. gigas*. This work provides a systemic characterization of the IIS and ILPR-mediated signaling pathways involved in the growth of *C. gigas*, which will provide valuable information toward our understanding of the molecular basis of growth regulation in oysters and other invertebrates.

## 2. Results

### 2.1. Identification of ILPR, IRS, IGFBP, and IGFALS in C. gigas

**Figure 1:**
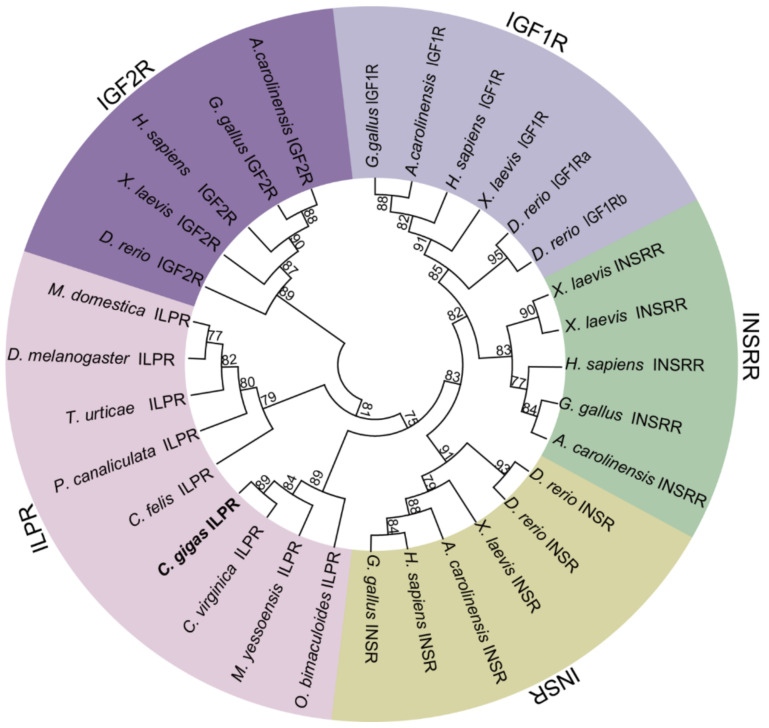
Phylogenetic analyses of the insulin receptors and insulin-like peptide receptors. The amino acid sequences of the insulin receptors and insulin-like peptide receptors from *Homo sapiens*, *Gallus gallus*, *Xenopus laevis*, *Anolis carolinensis*, *Danio rerio*, *Drosophila melanogaster*, *Musca domestica*, *Ctenocephalides felis*, *Mizuhopecten yessoensis*, *Tetranychus urticae, Octopus bimaculoid*, *Pomacea canaliculata*, *Crassostrea virginica,* and *Crassostrea gigas* were retrieved from the NCBI database. The phylogenetic tree was constructed using the neighbor-joining (NJ) approach in MEGA7. The reliability of the topological structure was tested using 1000 bootstrap replications.

**Figure 2:**
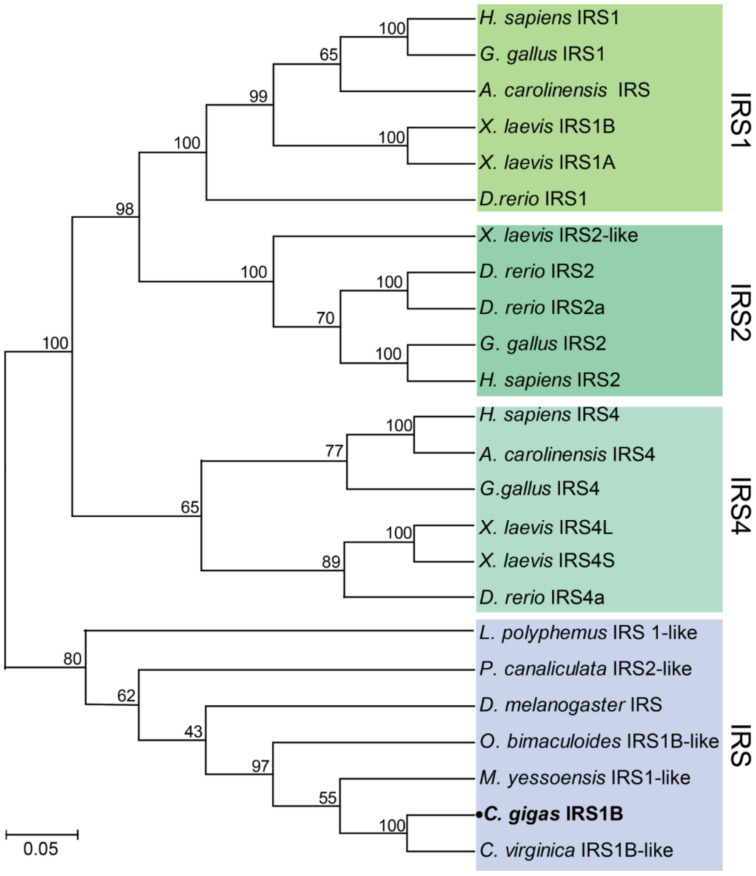
Phylogenetic analyses of the insulin receptor substrates. The amino acid sequences of the insulin receptor substrates from *Homo sapiens*, *Gallus gallus*, *Xenopus laevis*, *Anolis carolinensis*, *Danio rerio*, *Drosophila melanogaster*, *Limulus polyphemus*, *Mizuhopecten yessoensis*, *Octopus bimaculoid*, *Pomacea canaliculata*, *Crassostrea virginica,* and *Crassostrea gigas* were retrieved from the NCBI database. The phylogenetic tree was constructed using the neighbor-joining (NJ) approach in MEGA7. The reliability of the topological structure was tested using 1000 bootstrap replications.

**Figure 3:**
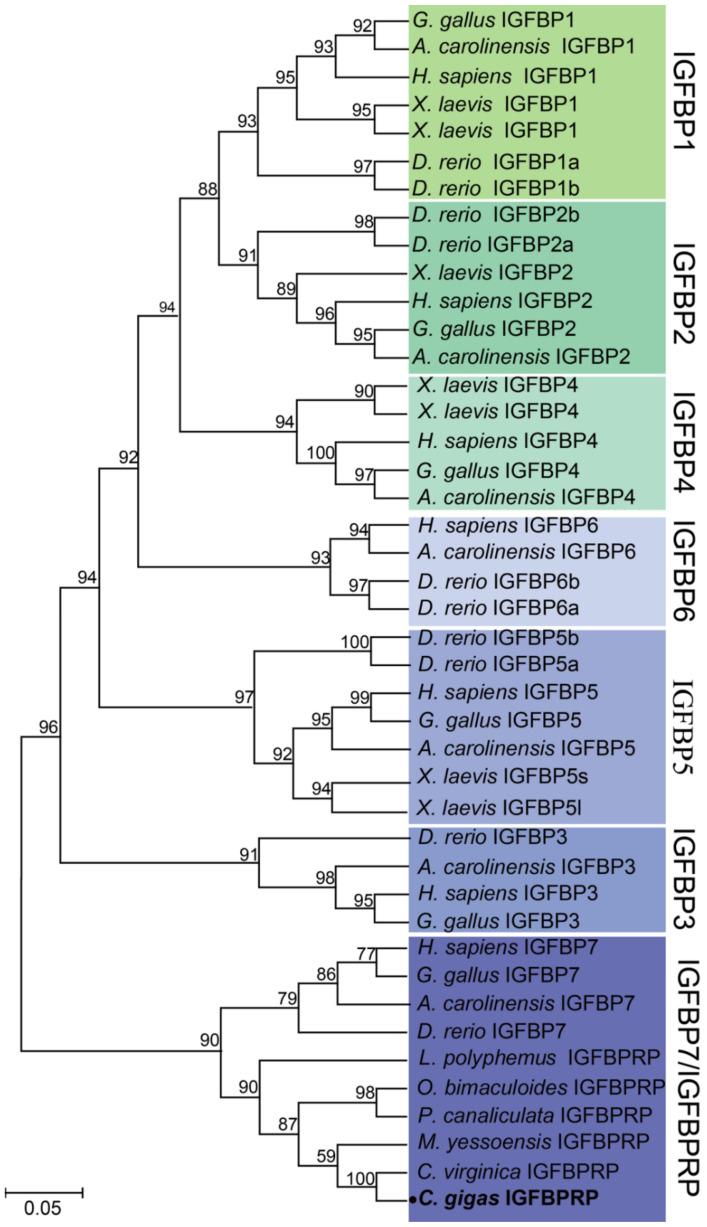
Phylogenetic analyses of the IGFBP superfamily. The amino acid sequences of the insulin-like growth factor-binding proteins from *Homo sapiens*, *Gallus gallus*, *Xenopus laevis*, *Anolis carolinensis*, *Danio rerio*, *Limulus polyphemus*, *Mizuhopecten yessoensis*, *Octopus bimaculoid*, *Pomacea canaliculata*, *Crassostrea virginica,* and *Crassostrea gigas* were retrieved from the NCBI database. The phylogenetic tree was constructed using the neighbor-joining (NJ) approach in MEGA7. The reliability of the topological structure was tested using 1000 bootstrap replications.

One *ILPR*, one *IRS*, one *IGFBP*, and seven *IGFALS* genes were identified in *C. gigas* through extensive genome and transcriptome data mining. The gene names, sequence characteristics, and accessions are provided in Table 1. Phylogenetic analysis showed that the *C. gigas* ILPR was clustered into one clade with the ILPRs of arthropods and other mollusks, while the vertebrate-specific IGF1R, IGF2R, INSR, and INSRR were clustered into separate clades (Figure 1). In addition, the IRS of *C. gigas* was clustered into one clade with the IRS of arthropods and other mollusks, while the three IRSs in vertebrates—IRS1, IRS2, and IRS4—were clustered into other separate clades (Figure 2). The sole IGFBPRP in *C. gigas* was clustered into the clade with vertebrate IGFBP7 (also known as IGFBPRP1) and the IGFBPRP from other mollusks, while the other six vertebrate-specific IGFBP members were clustered into other separate clades (Figure 3).

**Figure 4:**
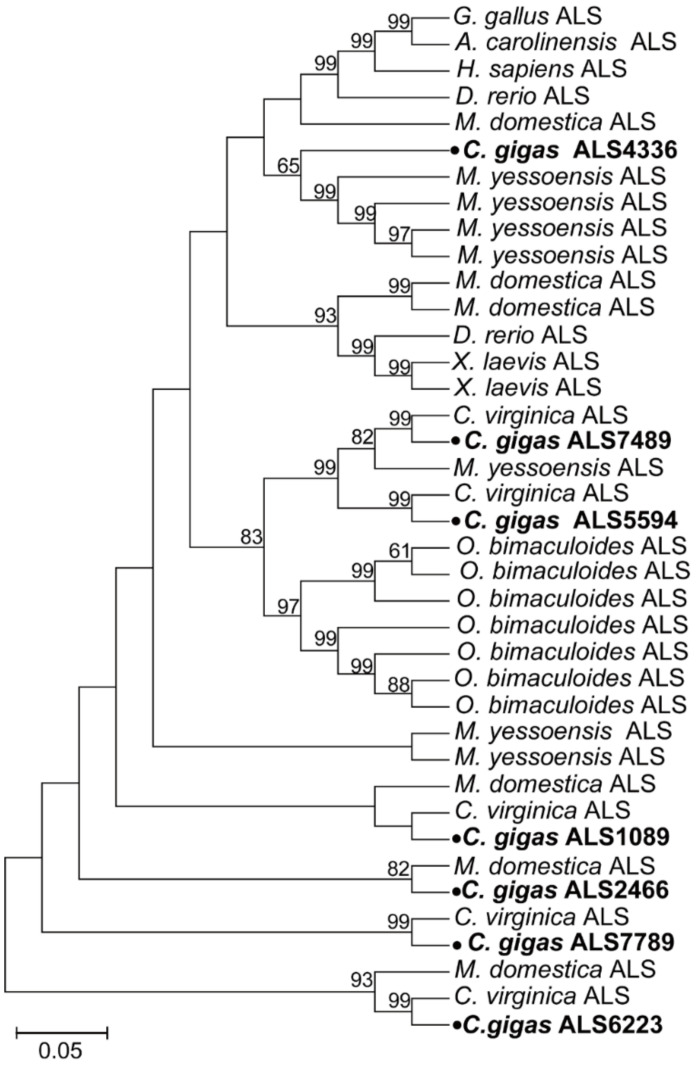
Phylogenetic analyses of the IGF ALSs. The amino acid sequences of the insulin-like growth factor-binding protein complex acid labile subunits from *Homo sapiens*, *Gallus gallus*, *Xenopus laevis*, *Anolis carolinensis*, *Danio rerio*, *Musca domestica, Mizuhopecten yessoensis*, *Octopus bimaculoid*, *Crassostrea virginica,* and *Crassostrea gigas* were retrieved from the NCBI database. The phylogenetic tree was constructed using the neighbor-joining (NJ) approach in MEGA7. The reliability of the topological structure was tested using 1000 bootstrap replications.

In striking contrast to the identification of only one IGFBP, seven IGFALSs were found in *C. gigas*. Phylogenetic analysis showed that the *C. gigas* ALS4336 was clustered into one clade with the ALSs of *Mizuhopecten yessoensis*, the ALS7489 and ALS5594 in *C. gigas* were clustered into one clade with the ALSs from *C. virginica*, *M. yessoensis*, and *Octopus bimaculoid*, and these three ALSs in *C. gigas* were clustered into a larger clade with the ALSs from several vertebrates. Furthermore, the ALS1089, ALS2466, ALS7789, and ALS6223 in *C. gigas* were clustered into one clade with the ALS of *C. virginica*, and had relatively closer homology to the ALS in invertebrates. The phylogenetic analysis also indicated that the ALS1089, ALS2466, ALS7789, and ALS6223 in *C. gigas* had a relatively primitive evolution status in comparison with the other three ALSs—ALS4336, ALS7489, and ALS5594 (Figure 4). 

### 2.2. Comparison of the Expression Profiles of the IIS, PI3K-AKT, and RAS-MAPK Signaling Pathway Genes between “Haida No.1” and Wild Oysters

**Figure 5:**
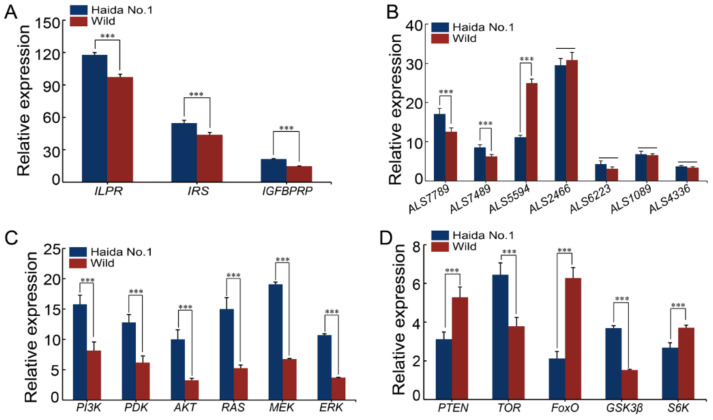
Expression profiles of the IIS, PI3K-AKT, RAS-MAPK, and TOR signaling pathway genes. (**A**) Expression profiles of the *ILPR*, *IRS*, and *IGFBPRP* between the “Haida No.1” and wild oysters. (**B**) Expression profiles of the *IGFALS* genes between the “Haida No.1” and wild oysters. (**C**) Expression profiles of the *PI3K*, *PDK*, *AKT*, *RAS*, *MEK*, and *ERK* genes between the “Haida No.1” and wild oysters. (**D**) Expression profiles of the *PTEN*, *TOR*, *FoxO*, *GSK3β,* and *S6K* genes between the “Haida No.1” and wild oysters. Data are expressed as the mean ± SD (n = 4). The significant difference among groups is indicated by the asterisk symbol *** (*p* < 0.05).

The IIS signaling pathway genes, including *ILPR*, *IGFBPRP,* and *IRS,* were all expressed at higher levels in fast-growing “Haida No.1” than in wild oysters (Figure 5A). Among the seven *ALS* genes, *ALS7789* and *ALS7489* were highly expressed in “Haida No.1”, while *ALS5594* was expressed at a higher level in wild oysters, and the other ALSs showed no expression difference between “Haida No.1” and wild oysters (Figure 5B).

The genes involved in the PI3K-AKT and RAS-MAPK signaling pathways, including *PI3K*, *PDK*, *AKT*, *RAS*, *MEK*, and *ERK*, were all highly expressed in “Haida No.1” (Figure 5C). Furthermore, the downstream genes of the PI3K-AKT signaling pathway, including *PTEN*, *TOR*, *FoxO*, *GSK3β,* and *S6K*, showed distinct expression profiles between the “Haida No.1” and wild oysters, with *GSK3β* and *TOR* being expressed at higher levels in “Haida No.1”, while *PTEN*, *FoxO,* and *S6K* were expressed at higher levels in the wild oysters (Figure 5D).

### 2.3. Effect of Nutrient Abundance on the IIS, PI3K-AKT, and RAS-MAPK Signaling Pathway Genes in C. gigas

**Figure 6:**
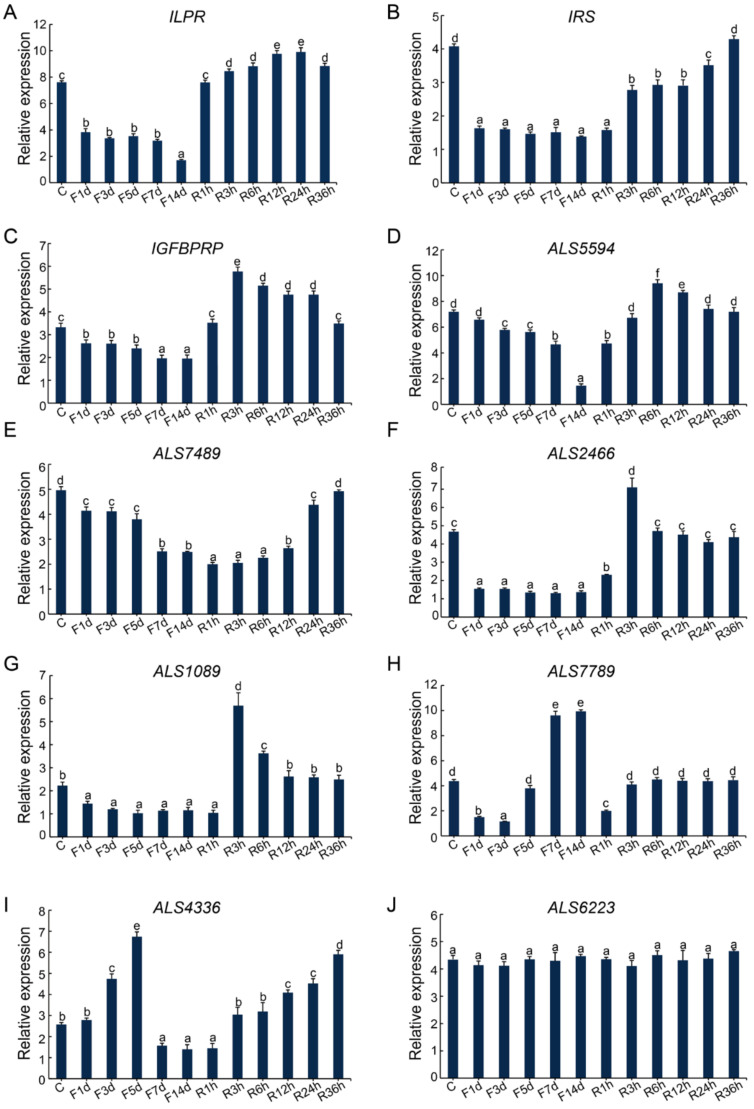
Effects of fasting and re-feeding on the expression of the IIS signaling pathway genes. C represents the control group; F1d, F3d, F5d, F7d, and F14d represent 1, 3, 5, 7, and 14 days after fasting, respectively; R1h, R3h, R6h, R12h, R24h, and R36h represent 1, 3, 6, 12, 24, and 36 h after re-feeding, respectively. (**A**) Effects of fasting and re-feeding on the expression of *ILPR*. (**B**) Effects of fasting and re-feeding on the expression of *IRS*. (**C**) Effects of fasting and re-feeding on the expression of *IGFBPRP*. (**D**) Effects of fasting and re-feeding on the expression of *ALS5594*. (**E**) Effects of fasting and re-feeding on the expression of *ALS7489*. (**F**) Effects of fasting and re-feeding on the expression of *ALS2466*. (**G**) Effects of fasting and re-feeding on the expression of *ALS1089*. (**H**) Effects of fasting and re-feeding on the expression of *ALS7789*. (**I**) Effects of fasting and re-feeding on the expression of *ALS4336.* (**J**) Effects of fasting and re-feeding on the expression of *ALS6223*. Data are expressed as the mean ± SD (n = 3). Significant differences (*p* < 0.05) among the timepoints of fasting and re-feeding are indicated by the values with different letters. At all timepoints, the lowercase letter “a” represents the lowest expression level, and is significantly different from “b”, as well as other lowercase letters such as “c”, “d”, etc.

The expression profiles of the *ILPR, IRS,* and *IGFBPRP* genes were quite distinct in the fasting and re-feeding culture experiment (Figure 6A–C). Specifically, *ILPR*, *IRS,* and *IGFBP* were significantly downregulated from day 1 to day 14 during the fasting treatment. After re-feeding, the expression of *ILPR* was significantly upregulated in 1 h, peaked at 12 h and 24 h, then decreased to a relatively lower level at 36 h (Figure 6A); the expression of *IRS* was significantly upregulated at 3 h and peaked at 36 h (Figure 6B); while the expression of *IGFBPRP* was significantly upregulated in 1 h and peaked at 3 h, then decreased and remained at a regular level comparable to that of the control at 36 h (Figure 6C). 

The seven *IGFALS*s showed gene-specific expression patterns after the fasting and re-feeding treatment. The *ALS5594, ALS7489*, *ALS2466,* and *ALS1089* genes were all significantly downregulated from day 1 to day 14 during the fasting treatment and upregulated in 1, 3, or 12 h after re-feeding (Figure 6D–G). In addition, the expression of *ALS7789* was first downregulated from day 1 to day 3, then upregulated from day 5 to day 14 during the fasting process. After re-feeding, its expression was first downregulated, then upregulated during 1–3 h, and finally maintained at a control level (Figure 6H). The expression of *ALS4366* was significantly upregulated on day 3 and peaked on day 5, then decreased from day 7 to day 14 during the fasting process. After re-feeding, the expression of *ALS4366* was upregulated at 3 h and peaked at 36 h (Figure 6I). The expression of *ALS6223* was not affected by the fasting and re-feeding treatment (Figure 6J). 

**Figure 7:**
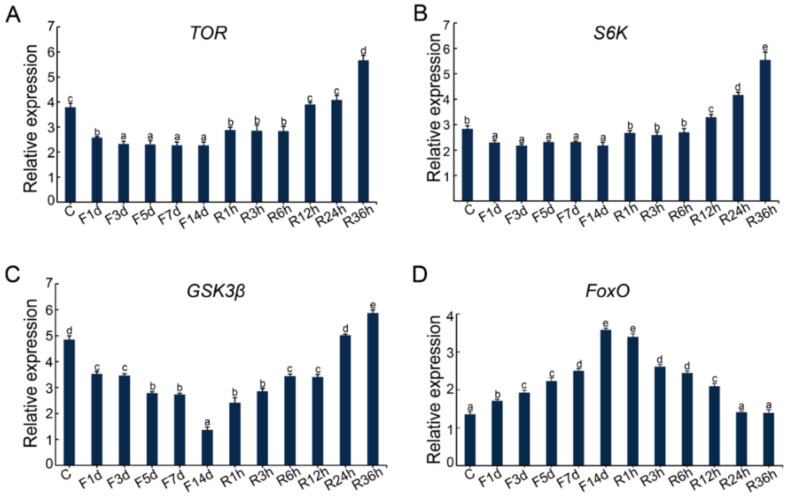
Effects of fasting and re-feeding on the expression of genes in the PI3K-AKT, RAS-MAPK, and TOR signaling pathways. C represents the control group; F1d, F3d, F5d, F7d, and F14d represent 1, 3, 5, 7, and 14 days after fasting, respectively; R1h, R3h, R6h, R12h, R24h, and R36h represent 1, 3, 6, 12, 24, and 36 h after re-feeding, respectively. (**A**) Effects of fasting and re-feeding on the expression of *TOR*. (**B**) Effects of fasting and re-feeding on the expression of *S6K*. (**C**) Effects of fasting and re-feeding on the expression of *GSK3β*. (**D**) Effects of fasting and re-feeding on the expression of *FoxO*. Data are expressed as the mean ± SD (n = 3). Significant differences (*p* < 0.05) among the timepoints of fasting and re-feeding are indicated by the values with different letters. At all timepoints, the lowercase letter “a” represents the lowest expression level, and is significantly different from “b”, as well as other lowercase letters such as “c”, “d”, etc.

The expression profiles of the *TOR, S6K, FoxO,* and *GSK3β* genes were quite distinct after the fasting and re-feeding treatment. The *TOR*, *S6K,* and *GSK3β* genes showed stepwise decrease from day 1 to day 14 during fasting. After re-feeding, those genes were significantly upregulated in 1 h and peaked at 36 h (Figure 7A–C), while the expression of *FoxO* was upregulated continuously from day 1 to day 14 during fasting, then downregulated from 3 h to 36 h to the control level after re-feeding (Figure 7D). 

### 2.4. Effect of Temperature on the IIS, PI3K-AKT, and RAS-MAPK Signaling Pathway Genes in C. gigas

**Figure 8:**
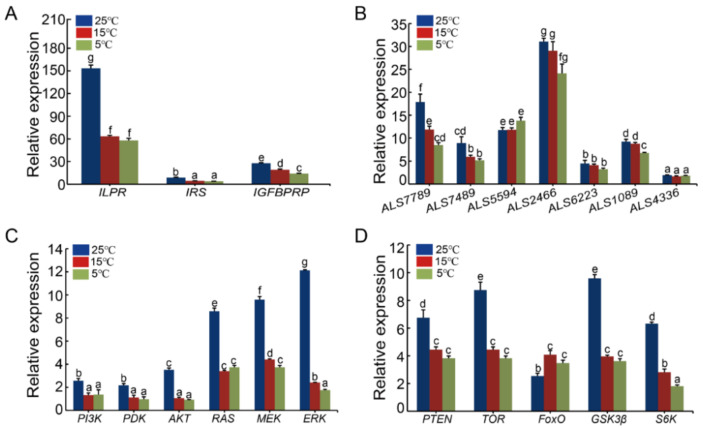
Effects of low temperature on the expression of the IIS, PI3K-AKT, RAS-MAPK, and TOR signaling pathway genes. (**A**) Effects of low temperature on the expression of *ILPR*, *IRS*, and *IGFBPRP.* (**B**) Effects of low temperature on the expression of *seven ALS.* (**C**) Effects of low temperature on the expression of *PI3K*, *PDK*, *AKT*, *RAS*, *MEK*, and *ERK* genes. (**D**) Effects of low temperature on the expression of *PTEN*, *TOR*, *FoxO*, *GSK3β,* and *S6K* genes. Data are expressed as the mean ± SD (n = 3). Significant differences (*p* < 0.05) among groups (25 ℃, 15 ℃, and 5 ℃) are indicated by the values with different letters. For all groups, the lowercase letter “a” represents the lowest expression level, and is significantly different from “b”, as well as other lowercase letters such as “c”, “d”, etc.

Low temperature significantly suppressed the growth of oyster larvae as well as the expression of the four insulin-like peptide genes [40]. Similarly, we found that the expression of the major IIS, PI3K-AKT, and RAS-MAPK signaling pathway genes, including the *ILPR*, *IRS*, *IGFBPRP*, *ALS7789*, *ALS7489*, *ALS1089*, *PI3K*, *PDK*, *AKT*, *RAS*, *MEK*, *ERK*, *PTEN*, *TOR*, *GSK3β,* and *S6K* genes, were also suppressed under the low culture temperature (Figure 8A–C). In contrast, the expression levels of the *ALS5594*, *ALS2466*, *ALS6223,* and *ALS4336* genes were not affected (Figure 8B), while the *FoxO* gene was expressed at a relatively higher level under low temperature than under normal temperature (Figure 8D). 

## 3. Discussion

The involvement of insulin/insulin-like growth factor (IIS) signaling pathways in regulating growth and energy metabolism has been well-studied in vertebrates, while the role of IIS pathways in the growth regulation of marine invertebrates, such as oysters, remains largely unexplored. In a previous study, we identified four insulin-like peptide genes in *C. gigas*, and showed that their expression levels were greatly associated with growth. How the insulin-like peptides function to play roles in the growth regulation of *C. gigas* deserves further investigation. In the present study, we performed extensive bioinformatics data mining and identified one *ILPR*, one *IRS*, one *IGFBP*, and seven *IGFALSs* genes in *C. gigas*. Phylogenetic analysis confirmed their identities and reconstructed their evolutionary relationships. Expression profiling of the major genes of the IIS and ILPR-mediated signaling pathways, including the PI3K-AKT and RAS-MAPK signaling pathways, showed that they were all expressed at higher levels in the fast-growing “Haida No.1” oysters. Furthermore, the expression levels of the IIS, PI3K-AKT, RAS-MAPK, and TOR signaling pathway genes were significantly affected by nutrient abundance and temperature in a gene-specific manner. These results suggest a critical role for the IIS signaling pathway in the growth regulation of *C. gigas*, and confirm the limited role of the IIS and ILPR-mediated signaling pathways in growth control in various organisms. 

Identification of the IIS, PI3K-AKT, and RAS-MAPK signaling pathway genes in *C. gigas* suggested a limited role for the IIS in growth and metabolism regulation. Phylogenetic analysis showed that the sole *C. gigas* ILPR was clustered with the ILPRs from other invertebrates, and had a relatively closer homology with the insulin receptor (INSR) in comparison with other members of the IR subfamily in vertebrates—including the IGF1R, IGF2R, and INSRR—indicating the similar physiological functions of the ILPR in *C. gigas* and the insulin receptor in vertebrates. Notably, although four insulin-like peptide genes were found in *C. gigas*, there was only one ILPR, which was strikingly different from the case in vertebrates, deserving further investigations on the evolution of ligand and receptor binding in insulin-like peptides. The IRS, which could bind to the phosphorylated tyrosine residues of beta subunits of the ILPR, is involved in transducing the IIS signaling pathway within the cell [41,42]. In our present study, in contrast to vertebrates—which have three IRSs—only one IRS was identified in *C. gigas*, indicating specific binding of the IRS to beta subunits of ILPR in *C. gigas*. 

The IGFBP family is composed of 6 distinct types of IGFBP (IGFBP1–6) and 10 IGFBPRPs in vertebrates, which is evolutionarily ancient and conserved [9]. In invertebrates, there is no definite evidence indicating the existence of traditional IGFBP1–6, which could bind to IGF-I and IGF-II with higher affinity than the related proteins (IGFBPRP1 to IGFBPRP10) [43]. IGFBP7, also known as IGFBPRP1, is distinct from other low-affinity IGFBPRPs in that it can strongly bind to insulin [23], suggesting that IGFBP7 is likely to have distinct biological functions from other IGFBPs. In our present study, one IGFBPRP and seven ALSs were found in *C. gigas*, and the IGFBPRP was clustered into one clade with the IGFBPRPs from other mollusks and the IGFBP7 from vertebrates. Our analysis suggested that the *C. gigas* IGFBPRP had a similar evolutionary origin and physiological function to the IGFBP7 in vertebrates, supporting the idea that the IGFBPRP (IGFBP7) may be the most ancient gene among the IGFBP subfamily. Furthermore, the number of ALSs is only one or two in vertebrates, while there are usually five to seven IGFALSs in arthropods and mollusks. The obviously larger number of IGFALSs in invertebrates than in vertebrates indicates that some of the ALSs could have been lost during evolution after the divergence of invertebrates and vertebrates.

Higher expression levels of insulin-like peptide genes were associated with fast growth in *C. gigas*, as revealed in our previous study. In the present work, the expression profiles of the major genes in the IIS signaling pathway were further determined in order to confirm the systemic regulatory role of the IIS signaling pathway in the growth regulation of oysters. Our results showed that the major genes of the IIS signaling pathway, including ILPR, IRS, and IGFBPRP, were all expressed higher in the fast-growing “Haida No.1” than in wild oysters. However, the *C. gigas* ALSs showed distinct expression profiles. The ALSs have been reported to play an indispensable role in the growth regulation of vertebrates through the GH/IGF system [44,45,46]. In our present study, the *ALS5594* gene was expressed higher in wild oysters, while the *ALS7789* and *ALS7489* genes were expressed at higher levels in the “Haida No.1” oysters, indicating that the ALS also played important but diverse roles in the growth regulation of *C. gigas*. 

**Figure 9:**
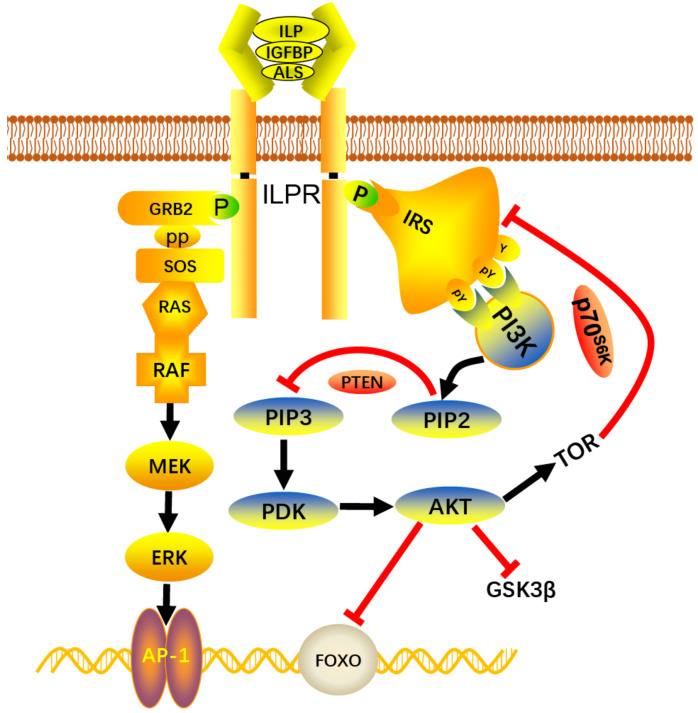
The proposed model of the role of the ILPR-mediated and IIS signaling pathways in the growth regulation of *C. gigas*. The binding of the insulin-like peptide to its receptor (ILPR) induces conformational changes in the alpha and beta subunits of the receptor. The IRS binds to the activated sites of the beta subunits and transduces signal to the cell through the PI3K-AKT and RAS-MAPK signaling pathways. The other elements, including PTEN, GSK3β, FoxO, and TOR, could regulate the activation of the PI3K-AKT signaling pathway in *C. gigas* through a similar mechanism as in vertebrates. The rapamycin-sensitive TORC1 (TOR complex 1) activates the translational regulator S6K (S6 kinase), leading to increased protein synthesis in the presence of nutrients, and the S6K could inhibit the activity of the IRS, finally regulating the PI3K-AKT and RAS-MAPK signaling pathways. Arrow (black line) and inhibitory sign (red line) indicate promotion and suppression, respectively.

In vertebrates, once the ternary complex “IGFs/insulins–IGFBP–ALS” binds to the receptor, the receptor is auto-phosphorylated and recruits the IRS; then, the IRS triggers different downstream signaling pathways, such as the PI3K-AKT and RAS-MAPK signaling pathways [47]. The MAPK and its downstream response elements are involved in cell growth and proliferation [48]. The activation of PI3K leads to the production of PIP3, which in turn promotes the activation of downstream factors such as AKT, TOR, and GSK3β to regulate cell proliferation, energy metabolism, and hormone secretion [49]. Recent studies have reported the involvement of several major members of the PI3K-AKT and RAS-MAPK signaling pathways in the regulation of adductor muscle growth in the triploid Pacific oyster [31,32]. In our present study, based on gene expression profiles, we found that the ILPR-mediated signaling pathway was conserved among vertebrates and invertebrates. The major genes of the PI3K-AKT and RAS-MAPK signaling pathways, including the *PI3K*, *PDK*, *AKT*, *RAS*, *MEK*, *ERK*, *TOR*, and *GSK3β* genes, were all expressed at higher levels in the “Haida No.1” oysters, while the negative regulators *PTEN*, *FoxO,* and *S6K* were all highly expressed in wild oysters. Therefore, ILPR is speculated to transduce signaling from the active ternary complex “ILP–IGFBP–ALS” to the downstream PI3K-AKT and RAS-MAPK signaling pathways, functioning to regulate energy metabolism and cell proliferation in oysters (Figure 9). 

Fasting/re-feeding and low-temperature culture experiments were performed in order to further confirm the hypothesis. In our present study, the major genes of the IIS signaling pathway—including the *ILPR*, *IRS*, *IGFBP*, and the seven *ALS* genes—all showed different expression patterns during the fasting and re-feeding process. The expression of the *ILPR*, *IRS*, *IGFBP*, *ALS5594*, *ALS7489*, *ALS2466,* and *ALS1089* genes universally decreased once food was stopped, and recovered to normal levels after re-feeding, which is consistent with the expression profile of the insulin-like peptide (*ILP*) gene in our previous study [40]. These results suggest that the ILP, ILPR, IRS, IGFBP, and ALSs may play systemic roles in food intake, nutrient digestion, and absorption through the ternary complex “ILP–IGFBPRP–ALS” in *C. gigas*. In addition, the expression of the *ALS7789* and *ALS4336* genes was upregulated during the fasting and re-feeding process, which is consistent with the expression profiles of the *MIRP3*, *MIRP3-like,* and *ILP7* genes in our previous study [40]. We speculate that the *ALS7789* and *ALS4336* genes may be associated with the physiological role of molluscan insulin-related peptide 3 (MIRP3), molluscan insulin-related peptide 3-like (MIRP3-like), and insulin-like peptide 7 (ILP7), and are indispensable in energy metabolism under the control of neuroendocrine activity. Furthermore, low temperature greatly suppressed the expression of the *ILPR*, *IRS*, *IGFBP*, *ALS7789*, *ALS7489*, and *ALS1089* genes. These results suggest that the IIS signaling pathway is responsive to fluctuations of nutrition condition and ambient temperature. 

The key elements of the PI3K-AKT and RAS-MAPK pathways also showed different expression patterns during the fasting and re-feeding process. The TOR nutrient pathway, which is regulated by insulin, nutrient abundance, and energy and growth factors, functions to modulate insulin-stimulated glucose transport, cellular metabolism, growth, and proliferation [50,51,52]. In addition, the TORC1 activates the translational regulator S6K (S6 kinase), leading to increased protein synthesis in the presence of nutrients [53]. In our study, the expression of the *TOR* and *S6K* genes was decreased during fasting, and recovered to normal levels after re-feeding. GSK3β phosphorylates the serine and threonine sites of a variety of substrates, including glycogen synthase, in order to regulate glycogen synthesis [54]. In our present study, the expression of *GSK3β* was inhibited under food deprivation conditions, and recovered to normal levels once food was abundant, indicating the reduction of glycogen synthesis under food deprivation conditions. As the negative regulator, FoxO regulates glucose and lipid metabolism through the transcriptional activation of certain genes [55]. In our study, the expression of *FoxO* was upregulated during fasting and downregulated after re-feeding. Furthermore, the expression of the IIS and ILPR-mediated signaling pathway genes was inhibited under low culture temperature, except for the negative regulator *FoxO*. These results confirm that the roles of the IIS and ILPR-mediated signaling pathways are limited, and that ILPR could transduce signaling from the active ternary complex “ILP–IGFBP–ALS” to the downstream PI3K-AKT and RAS-MAPK signaling pathways, regulating the energy metabolism and cell proliferation of *C. gigas* (Figure 9). 

## 4. Materials and Methods

### 4.1. Sequence Analysis

The amino acid sequences of the IIS signaling pathway genes—including ILPR, IRS, IGFBP and IGFALS—from Homo sapiens, Gallus gallus, Xenopus laevis, Anolis carolinensis, Danio rerio, Limulus polyphemus, Musca domestica, Tetranychus urticae, Aplysia californica, Mizuhopecten yessoensis, Octopus bimaculoid, Pomacea canaliculata, Crassostrea virginica, and Crassostrea gigas were retrieved from the NCBI database. Detailed information on these sequences is provided in supplementary Table S1. The sequences were aligned using ClustalW2 (http://www.ebi.ac.uk/Tools/msa/clustalw2/, accessed on 5 July 2020), and the phylogenetic trees were constructed using the neighbor-joining (NJ) approach in MEGA7 [56]. The reliability of the topological structure was tested using 1000 bootstrap replications.

### 4.2. Real-Time PCR and Statistical Analysis

All of the primers used for real-time PCR were designed using Primer Express software (Applied Biosystems, Foster, CA, USA), and are provided in Table 2. The two-fold dilutions of cDNA isolated from adductor muscle, and the internal control gene *elongation factor 1α* (E*F1α*), were used to assess primer efficiency, and the primer sets with an efficiency of 90–110% were used for real-time PCR analysis. The real-time PCR reactions were carried out in a LightCycler 480 machine (Roche, Basel, Switzerland) in a 20 μL system with a mixture of 10 μL 2×SYBR Premix ExTaq (Qiagen, Düsseldorf, Germany), 2.0 μL diluted cDNA or double-distilled water as a negative control, 6 μL PCR-grade water, and 1.0 μL of each primer (10 μM). The PCR reactions were initiated via denaturation at 95 °C for 3 min, followed by 40 amplification cycles at 95 °C for 15 s and at 60 °C for 30 s. Dissociation protocols were used to measure melting curves. The raw data of all the qRT-PCR were provided in supplementary Table S2. The relative expression level (RNA abundance) was calculated by dividing the copy number of the target gene by that of the internal control gene. Data were expressed as the mean ± SD. The one-way ANOVA and Student’s *t*-test were used to determine statistical differences for single or multiple comparisons, respectively. The statistical significance was set as *p* < 0.05, as indicated by the asterisk symbol or by values with different letters.

### 4.3. Expression Profiles of the Major Genes in the IIS, PI3K-AKT, and RAS-MAPK Signaling Pathways

The expression profiles of the major genes in the IIS, PI3K-AKT, and RAS-MAPK signaling pathways—including *ILPR*, *IRS*, *IGFBPRP*, *IGFALS*, *PI3K*, *PDK*, *AKT*, *RAS*, *MEK*, *ERK*, *PTEN*, *TOR*, *FoxO*, *GSK3β*, and *S6K*—were determined in the adductor muscles of “Haida No.1” and wild oysters. Total RNA was extracted using Trizol Reagent (Invitrogen, Carlsbad, CA, USA) and reversely transcribed into cDNA according to the PrimeScript™ RT reagent Kit with gDNA Eraser (Perfect Real Time) (TaKaRa, Japan), according to the manufacturer’s instructions. Real-time PCR was carried out as described in Section 4.2.

### 4.4. Fasting and Re-Feeding Culture Experiment

Ninety eight-month-old *C. gigas* were randomly divided into three groups and used for the fasting and re-feeding experiments. Six oysters collected before the experiment were used as controls (as indicated by C); the other oysters were starved for fourteen days and then re-fed with frozen *Chlorella* ad libitum for thirty-six hours. Samples were collected on days 1, 3, 5, 7, and 14 (as indicated by F1d, F3d, F5d, F7d, and F14d, respectively) during the fasting process, and at 1, 3, 6, 12, 24, and 36 h (as indicated by R1h, R3h, R6h, R12h, R24h, R36h, respectively) after re-feeding, with 6 oysters at each time point. Various tissues—including the labial palp, gill, mantle, digestive gland, hematocytes, heart, visceral ganglia, and adductor muscle—were rapidly excised, frozen in liquid nitrogen, and then stored at −80 °C until use. Total RNA extraction and cDNA synthesis were performed as described above. The cDNA from all eight tissues at each sampling point was pooled into one sample, with equal amounts per tissue, and used for real-time PCR analysis according to the method described in Section 4.3. 

### 4.5. Low Temperature Culture Experiment

Oyster umbo larvae of similar sizes (2.5 ± 0.3 mm) were stocked into 3 75 L cylindrical polyethylene vessels (larval density of 4/mL) and reared at 5 ℃, 15 ℃, and 25 ℃, respectively. Light was supplied by fluorescent lamps with 12:12 Light: Dark photoperiods. For each culture temperature, 3 random replicates were allocated, the survival and feeding situation of the larvae was monitored daily for 10-day culture, the shell height of 30 randomly collected larvae was measured, and the whole larvae were collected and deposited into RNAstore (CWBIO, Beijing, China) until used for RNA expression analyses. 

## 5. Conclusions

In summary, we identified and characterized one *ILPR*, one *IRS*, one *IGFBPRP*, and seven *IGFALSs* in *C. gigas*. Expression profiling of these genes in fast-growing oysters and in slow-growing wild oysters suggested their critical roles in growth regulation. Nutrient abundance and ambient temperature greatly affected the expression profiles of these genes. Furthermore, the major genes of the ILPR-mediated PI3K-AKT and RAS-MAPK signaling pathways were all expressed at higher levels in the fast-growing “Haida No.1” oysters, and were significantly suppressed under low-temperature conditions. These observations suggest that the IIS signaling pathway is involved in the growth regulation of *C. gigas* through regulating food intake, nutrition metabolism, and cell proliferation. This work provides valuable information for further investigation on growth regulation mechanisms in mollusks, as well as other invertebrates.

## Figures and Tables

**Table 1:** Sequence characteristics of the IIS signaling pathway genes in *Crassostrea gigas.*

Gene Name	Gene Full Name	Amino Acid (aa)	mRNA (bp)	ORF (bp)	NCBI Gene ID
*ILPR*	Insulin-like peptide receptor	1561	9092	4686	105348544
*IRS*	Insulin receptor substrate 1-B	1400	6375	4203	105342662
*IGFBPRP*	Insulin-like growth factor-binding protein-related protein 1	258	1926	777	105339347
*ALS4336*	Insulin-like growth factor-binding protein complex acid labile subunit	858	3189	2577	105331921
*ALS7489*	Insulin-like growth factor-binding protein complex acid labile subunit	1000	3987	3003	105342288
*ALS5594*	Insulin-like growth factor-binding protein complex acid labile subunit	978	4175	2937	105319673
*ALS1089*	Insulin-like growth factor-binding protein complex acid labile subunit	456	4620	1371	105324304
*ALS2466*	Insulin-like growth factor-binding protein complex acid labile subunit	913	4713	2744	105338877
*ALS7789*	Insulin-like growth factor-binding protein complex acid labile subunit	780	2607	2343	105342519
*ALS6223*	Insulin-like growth factor-binding protein complex acid labile subunit	349	1726	1050	105320119

**Table 2:** Primers used for the real-time PCR in this study.

Gene Name	Forward Primer (5′-3′)	Reverse Primer (5′-3′)
*ILPR*	ACCAGGGACTGTCCAATGAG	GGTCTGTAGCGCCAACATTT
*IRS*	TGTGGGTCAGGAAGGGAATC	CCAAACGAGCCTGACCTAGA
*IGFBPRP*	ACCTCGCCTGTAAGATGGAC	TCGGTACCACAGAGTGTGTC
*ALS5594*	GGCCGCTTATGCTAATGGAG	ACTCGTCCAACACATCCTGT
*ALS7789*	GTCTCCCGAGGGACATACTG	GTGCAACGTCTGAATCTCGT
*ALS7489*	TTCCTCGAGAACACGGACAT	AGGTGTTGGAAGGTGTAGGG
*ALS2466*	AACAGGCACTACCCAAGGTT	TAGAGGAAGCGGTGGTTTGT
*ALS6223*	CGAGCGTCAAAGATGTCCTG	TCGATCCCGATCCGTTTGAT
*ALS1089*	TCAACAACACAGCGTGCTTA	CATTTGAAAGCGGTGCCATC
*ALS4336*	CCACAGAACGCCTTTGAGAG	GGGAACTGCTGAACGAACTC
*PI3K*	TCCGTTCACATACCAAACGC	GGGCAGAGTGGCTTCATAGA
*PDK*	CATCAAGGTGCTGGAGAAGC	CTGTCTGTGTCCTGGAAGGT
*AKT*	AGAGGTTGCTCCAATCGTCA	ATGAAACACGCCATCAGCTC
*GSK3β*	CTAGCCTACATCCACTCGCA	AGGAGCCCTGTAGTAACGTG
*mTOR*	ACGTGACAAGACCTCCACAT	CAGGATCCCGGGAAGGTATC
*FoxO*	TCGCTACTACAGCGTTGTCT	GCCTGTCGTAGAAGCGAATC
*PTEN*	ACAAGATGGCGGATGTTGTG	GGGTTGTGGTCATCGAATGG
*RAS*	GACCCGACCATAGAGGACAG	TCCCTGCCCATTCTTCATGT
*MAPK*	TGGCTGTATCCTGGCAGAAA	TGTTCCATGGGACTTTGGGT
*ERK*	GCAATGGATCCACCACTTCC	ACGTGTTACCATGCACACTG
*EF1α*	AGTCACCAAGGCTGCACAGAAAG	TCCGACGTATTTCTTTGCGATGT

## Supplementary Materials

The following are available online at https://www.mdpi.com/article/10.3390/ijms22105259/s1.

## Abbreviations

IIS	Insulin/insulin-like growth factor (IIS) signaling
IRS	Insulin receptor substrate
IGFBPRP	Insulin-like growth factor-binding protein-related protein 1
IGFALS	Insulin-like growth factor-binding protein complex acid labile subunits
ILPR	Insulin-like peptide receptor
PI3K	Phosphoinositide 3-kinase
AKT	Protein kinase B
TOR	target of rapamycin
AMPK	monophosphate-activated protein
